# Comment on “Tapered Cuff versus Conventional Cuff for Ventilator-Associated Pneumonia in Ventilated Patients: A Meta-Analysis of Randomized Controlled Trials”

**DOI:** 10.1155/2019/2679513

**Published:** 2019-07-07

**Authors:** Bert Maertens, Stijn Blot

**Affiliations:** ^1^Ghent University, Department of Internal Medicine, De Pintelaan 185, 9000 Ghent, Belgium; ^2^University of Queensland, Burns Trauma and Critical Care Research Centre, Level 9, UQ Health Sciences Building, Royal Brisbane and Women's Hospital, Herston, QLD 4029, Australia

Despite progress in the field of infection prevention, avoiding ventilator-associated pneumonia (VAP) remains challenging. In this regard, we read with great interest the recently published paper by Huang et al. and would like to compliment the authors on this interesting meta-analysis [1].

Innovations in endotracheal tube design have emerged in an effort to avoid the microaspiration of contaminated oropharyngeal secretions, the main pathogenic mechanism for pneumonia development. Most of these innovations show promising results in laboratory settings, but often fail to translate this to clinically important benefits. While ultrathin polyurethane (PU) cuffs and taper-shaped cuffs are capable of reducing microaspiration, they fail to reduce pneumonia incidence in long-term ventilated patients [1–3]. We believe this is because a better sealing cuff leads to overabundant accumulation of subglottic secretions. In combination with short episodes of underinflation, this may lead to massive microaspiration, thereby nullifying any effect of temporarily improved sealing. Accordingly, both subglottic secretion drainage (SSD) and continuous cuff pressure regulation have shown to be effective in pneumonia prevention [4, 5].

The overall findings of Huang et al. are similar to those we found earlier [1, 3]. However, we were surprised to see data differed significantly between our review and the one by Huang et al. (Table 1). Two of these differences are due to a different search strategy. While our review included two studies without full publication [6, 7], Huang et al. did not search for unpublished work. The third difference is the data reported for the study by Philippart et al. [8]. They compared four groups; PU tapered, polyvinylchloride (PVC) tapered, PU cylindrical, and PVC cylindrical cuffs. We chose to combine both tapered groups and both cylindrical groups, while Huang et al. chose only to consider the PVC groups.

We argue, however, that the last two differences are, in our opinion, due to erroneous comparisons by Huang et al. First of all, we believe the data reported for the study by Monsel et al. are wrong [9]. Huang et al. seem to have used the number of second postoperative pneumonia episodes, instead of the total number of microbiologically confirmed pneumonia episodes, as we did. Secondly, Huang et al. include a study by Mahmoodpoor et al. that was not included in our analysis [10]. We excluded this study because the two endotracheal tubes that were compared differed not only with regard to the shape of the cuff. In the tapered cuffed tubes, SSD was applied while this was not the case in the standard cuffed tubes. Since there is convincing evidence that SSD reduces VAP incidence, we believe that the difference observed in the study is largely attributable to SSD [5]. Not unexpectedly, this study is the only one of the five studies included in the meta-analysis of Huang et al. that found a significant difference in VAP incidence between both groups.

Nevertheless, we agree with the authors that there is no evidence that taper-shaped cuffs provide any benefit on clinically important outcomes. However, as highlighted above, we believe this might be due to accumulation of secretions above a better sealing cuff. The effect of taper-shaped cuffs vs. standard cuffs with concomitant use of SSD and/or continuous cuff pressure regulation remains largely unexplored.

## Figures and Tables

**Table 1:** Differences between data reported in the review of Huang et al. vs. that of Maertens et al.

Study	Huang et al.				Maertens et al.				Reason for difference
	Tapered		Standard		Tapered		Standard		
	E	T	E	T	E	T	E	T	
Bent et al.	Not included				0	37	1	43	Unpublished study
Mahmoodpoor et al., 2013	6	32	7	32	6	32	7	32	No difference
Saito et al.	Not included				23	106	23	106	Unpublished study
Philippart et al.	17	129	14	129	33	162	38	163	Huang et al. only considered PVC cuffed tubes
Monsel et al.	10	52	8	57	15	52	16	57	Data error
Jailette et al.	33	162	38	163	33	162	38	163	No difference
Mahmoodpoor et al., 2017	30	138	46	138	Not included				SSD applied in tapered group

E: events; T: total; PVC: polyvinylchloride; SSD: subglottic secretion drainage.
